# pH regulatory divergent point for the selective bio-oxidation of primary diols during resting cell catalysis

**DOI:** 10.1186/s13068-022-02171-5

**Published:** 2022-06-30

**Authors:** Xia Hua, ChenHui Zhang, Jian Han, Yong Xu

**Affiliations:** 1 Key Laboratory of Forestry Genetics & Biotechnology (Nanjing Forestry University), Ministry of Education, Nanjing, 210037 People’s Republic of China; 2 Jiangsu Co-Innovation Center of Efficient Processing and Utilization of Forest Resources, College of Chemical Engineering, Nanjing Forestry University, No. 159 Longpan Road, Nanjing, 210037 People’s Republic of China; 3 Jiangsu Province Key Laboratory of Green Biomass-Based Fuels and Chemicals, Nanjing, 210037 People’s Republic of China

**Keywords:** Hydroxyl acid, Whole-cell catalysis, Oxidation, Sealed-oxygen supply, pH regulation

## Abstract

**Background:**

Hydroxyl acid is an important platform chemical that covers many industrial applications due to its dual functional modules. At present, the traditional technology for hydroxyl acid production mainly adopts the petroleum route with benzene, cyclohexane, butadiene and other non-renewable resources as raw materials which violates the development law of green chemistry. Conversely, it is well-known that biotechnology and bioengineering techniques possess several advantages over chemical methods, such as moderate reaction conditions, high chemical selectivity, and environmental-friendly. However, compared with chemical engineering, there are still some major obstacles in the industrial application of biotechnology. The critical issue of the competitiveness between bioengineering and chemical engineering is products titer and volume productivity. Therefore, based on the importance of hydroxyl acids in many fields, exploring a clean, practical and environmental-friendly preparation process of the hydroxyl acids is the core purpose of this study.

**Results:**

To obtain high-purity hydroxyl acid, a microbiological regulation for its bioproduction by *Gluconobacter oxydans* was constructed. In the study, we found a critical point of chain length determine the end-products. *Gluconobacter oxydans* catalyzed diols with chain length ≤ 4, forming hydroxyl acids, and converting 1,5-pentylene glycol and 1,6-hexylene glycol to diacids. Based on this principle, we successfully synthesized 75.3 g/L glycolic acid, 83.2 g/L 3-hydroxypropionic acid, and 94.3 g/L 4-hydroxybutyric acid within 48 h. Furthermore, we directionally controlled the products of C5/C6 diols by adjusting pH, resulting in 102.3 g/L 5‑hydroxyvaleric acid and 48.8 g/L 6-hydroxycaproic acid instead of diacids. Combining pH regulation and cell-recycling technology in sealed-oxygen supply bioreactor, we prepared 271.4 g 5‑hydroxyvaleric acid and 129.4 g 6-hydroxycaproic acid in 6 rounds.

**Conclusions:**

In this study, a green scheme of employing *G. oxydans* as biocatalyst for superior-quality hydroxyl acids (C2–C6) production is raised up. The proposed strategy commendably demonstrated a novel technology with simple pH regulation for high-value production of hydroxyl acids via green bioprocess developments.

**Supplementary Information:**

The online version contains supplementary material available at 10.1186/s13068-022-02171-5.

## Introduction

Consumption of traditional petrochemical resources leads to environmental pollution, causing toxicity, carcinogenicity, and biological aggregation of harmful chemical substances. Moreover, excessive use of coal, oil and natural gas makes the non-renewable resources on earth increasingly scarce [1, 2]. Hence, to overcome the dilemma of insufficient resources and low utilization, green synthesis methods have been developed. As results, these environmentally friendly economics has become an important technology impetus in recent years [3, 4]. Consequently, green methods for amino acids, vitamins, polymers, and other chemicals production have been recently reported [5, 6].

Hydroxyl acid (HOCH_2_–(CH_2_)_n_–COOH) is an important bioresource intermediates containing both hydroxyl and carboxyl groups at terminal positions [7, 8]. Due to the unique properties of dual-functional module, hydroxyl acids can be converted to various fine chemical intermediates and biopolymer precursors [9–11]. Especially, because of the excellent biocompatibility and biodegradability, they have become the spotlight of the medical polymers industry [12–14]. However, the existing hydroxyl acid production technology, oxidation or reduction via chemical catalysts or biocatalysts, has low yield and high environmental toxicity [15–17]. In chemical catalysis, serious by-products are generated due to the redox relationship between hydroxyl and carboxyl groups [18]. For example, the most common method for the synthesis of glycolic acid (GA) in the industry is chloroacetic acid or hydroxy acetonitrile hydrolysis [19]. However, the raw materials applied in two processes are highly toxic and corrosive, causing environmental pollution and safety issues. Moreover, the preparation of hydroxy acids by fermentation is also a hot research field, although fermentation technology has not been adopted in industry. At present, many hydroxyl acids have been successfully synthesized by employing recombinant microorganisms such as *Escherichia coli* [20, 21], *Klebsiella pneumoniae* [22, 23]*,* and *Corynebacterium glutamicum* [24, 25]. However, fermentation technology has some inherent disadvantages of high cost and long reaction cycle, which limits its application in large-scale industrial production [26]. Therefore, the development of a green and environmental-friendly technology is required for preparing high-quality hydroxyl acids.

Biocatalysis, a promising approach for sustainable development of industry in the future, has applied in various fields, including food, chemical, medicine, environmental, and energy [27, 28]. Hence, in-depth development and application of biocatalysis technology in the production of hydroxyl acids would provide a promising direction for the industrial production. *Gluconobacter oxydans* (*G. oxydans*) is a Gram-negative bacterium known for its incomplete oxidation capability, attributed to the membrane-bound dehydrogenases [29, 30] such as alcohol [31], aldehyde [32], glycerol [33], and sorbitol dehydrogenases [34]. These membrane-bound dehydrogenases are mainly responsible for catalyzing alcohols and aldehydes to corresponding acids and ketones. Furthermore, membrane-bound dehydrogenases are directly located on the cell membrane, and substrates are oxidized into products and released into the periplasm without carrier transport, considerably improving the catalytic efficiency [35]. These properties enabled *G. oxydans* to be employed as a common industrial biocatalyst for the industrial production of vitamin C [36], xylonic acid [37], and gluconic acid [38]. In addition, glycolic acid, furoic acid, 2-hydroxyacetone, and other platform compounds are also preliminarily synthesized on an industrial scale [39, 40]. These special characteristics, coupled with strict regioselectivity and stereoselectivity of *G. oxydans*, have facilitated the large-scale production of hydroxyl acids. In addition, depending on *G. oxydans*, Sang-Hyun et al. have realized the bio-preparation of 6-hydroxycaproic acid (6-HCA) recently. During 100 h continuous whole-cell catalysis, about 350 mg 6-HCA was selectively produced in 10 mL broth with a fed-batch mode (35 g/L) [41]. However, the low productivity (0.35 g/L/h) obviously could not satisfy the demand of large-scale production. Therefore, this study was committed to combination of sealed oxygen-supplied technology and pH regulation to realize the efficient preparation of hydroxyl acid covering C2–C6.

Considering the bottlenecks in the industrial-scale preparation of hydroxyl acids, it is important to develop an efficient and green method by investigating the reaction mechanism of *G. oxydans* whole-cell catalysis of primary diols for hydroxyl acids production. As results, combined SOS technology, we successfully achieved high-titer of GA, 3-hydroxypropionic acid (3-HPA), and 4-hydroxybutyric acid (4-HBA). Furthermore, a green route for whole-cell catalysis can precisely control the production of 5‑hydroxyvaleric acid (5-HVA) and 6-HCA by pH regulation.

## Materials and methods

### Materials

Ethylene glycol (EG), 1,3-propylene glycol (1,3-PG), 1,4-butylene glycol (1,4-BG), 1,5-pentylene glycol (1,5-PG), 1,6-hexylene glycol (1,6-HG), GA, 3-HPA, 4-HBA, 5-HVA, 6-HCA, glutaric acid (GTA) and adipic acid (AA) were obtained from Aladdin Chemical Reagent Corporation (China). Yeast Extract was purchased from Sigma-Aldrich. Sorbitol, MgSO_4_, KH_2_PO_4_, K_2_HPO_4_, (NH4)_2_SO_4,_ and CaCO_3_ were obtained from Sinopharm Chemical Reagent Co., Ltd. (China). All other chemicals were of analytical grade and were commercially available.

### Microorganism

*Gluconobacter oxydans* NL71 was isolated from *G. oxydans* ATCC 621 using the crude lignocellulosic hydrolysate domestication process. The strain was preserved in sorbitol–agar medium containing 50 g/L sorbitol, 5 g/L yeast extract, and 15 g/L agar, at 4 ℃. The inoculum was cultivated in an Erlenmeyer flask at 30 ℃ for 24–36 h, with continuous agitation at 220 rpm using a mechanical shaker (New Brunswick Scientific). The nutrient medium was composed of 100 g/L sorbitol and 10 g/L yeast extract. Cultured cells were centrifuged at 6000 rpm at 4 ℃, for 5–10 min in a freezing centrifuge (Avanti J-26 XP, Beckman Coulter). Centrifuged cells were washed 3 times with sterile saline and sterile water, respectively [42].

### Whole-cell catalysis

**Fig. 1:**
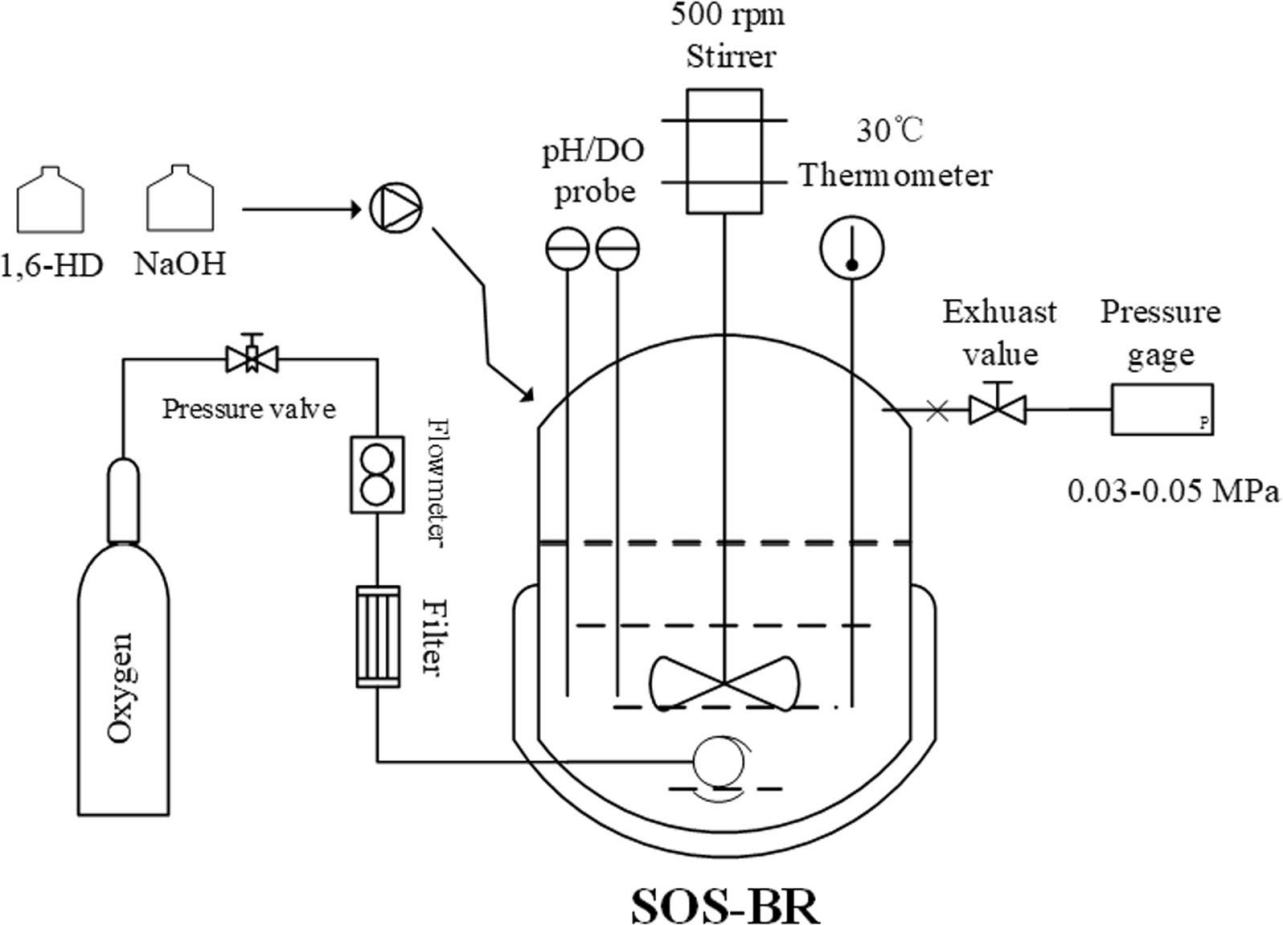
Bioreactor operation model of sealed oxygen-supplied bioreactor (SOS-BR) for the whole-cell catalysis

Bioprocess for whole-cell catalysis of 1,6-HG was carried out in a 3-L bioreactor with 1 L broth containing *G. oxydans* with OD_600_ = 10, 5 g/L yeast extract as the nitrogen source, and 0.5 g/L MgSO_4_, 1 g/L KH_2_PO_4_, 2 g/L K_2_HPO_4_, and 5 g/L (NH_4_)_2_SO_4_ as nutrients. Biocatalysis temperature was maintained at 30 °C and a stirring speed of 500 rpm. In addition, as *G. oxydans* is an obligate microorganism, oxygenation (purity ≥ 99.9) was maintained throughout the bioprocess. However, excessive oxygenation not only increases the cost but also leads to the wastage of resources due to low oxygen utilization. Considering that the whole-cell catalysis by *G. oxydans* is resting-cell catalysis without generating waste gas such as CO_2_. Hence, we employed a sealed oxygen-supplied bioreactor (SOS-BR), as shown in Fig. 1, for whole-cell catalysis. The pressure of the reactor was controlled at 0.03–0.05 MPa [43] and the pH of broth was adjusted by 30% NaOH. For cell-cycling technology, *G. oxydans* was harvested via centrifugation at 6000–8000 rpm for 5 min under 4 °C and cell pellets were washed with deionized water. Finally, the bacteria were re-inserted into the fresh medium for a new round of whole-cell catalysis.

### Analytical methods

The titer of EG, 1,3-PG, 1,4-BG, 1,5-PG, 1,6-HG, hydroxyl acids and diacids was detected by high-performance liquid chromatography (HPLC, Agilent 1260 Series) equipped with a differential detector. The column used for separation was the Aminex Bio-Rad HPX-87H and 5 mM H_2_SO_4_ was used as mobile phase at a flow rate of 0.6 mL/min. Moreover, the yield of hydroxyl acid was calculated according to Formula 1 as follows:

1 $$y = \frac{{{\text{C}}2{\text{*M}}1}}{{{\text{C}}1{\text{*M}}2}}*100\%$$

Due to the difference in experiment data, three parallel assays were performed for each experiment to ensure the reliability of results.

## Results and discussion

### The whole-cell catalysis of primary diols (C2–C6) by ***G. oxydans***

**Fig. 2:**
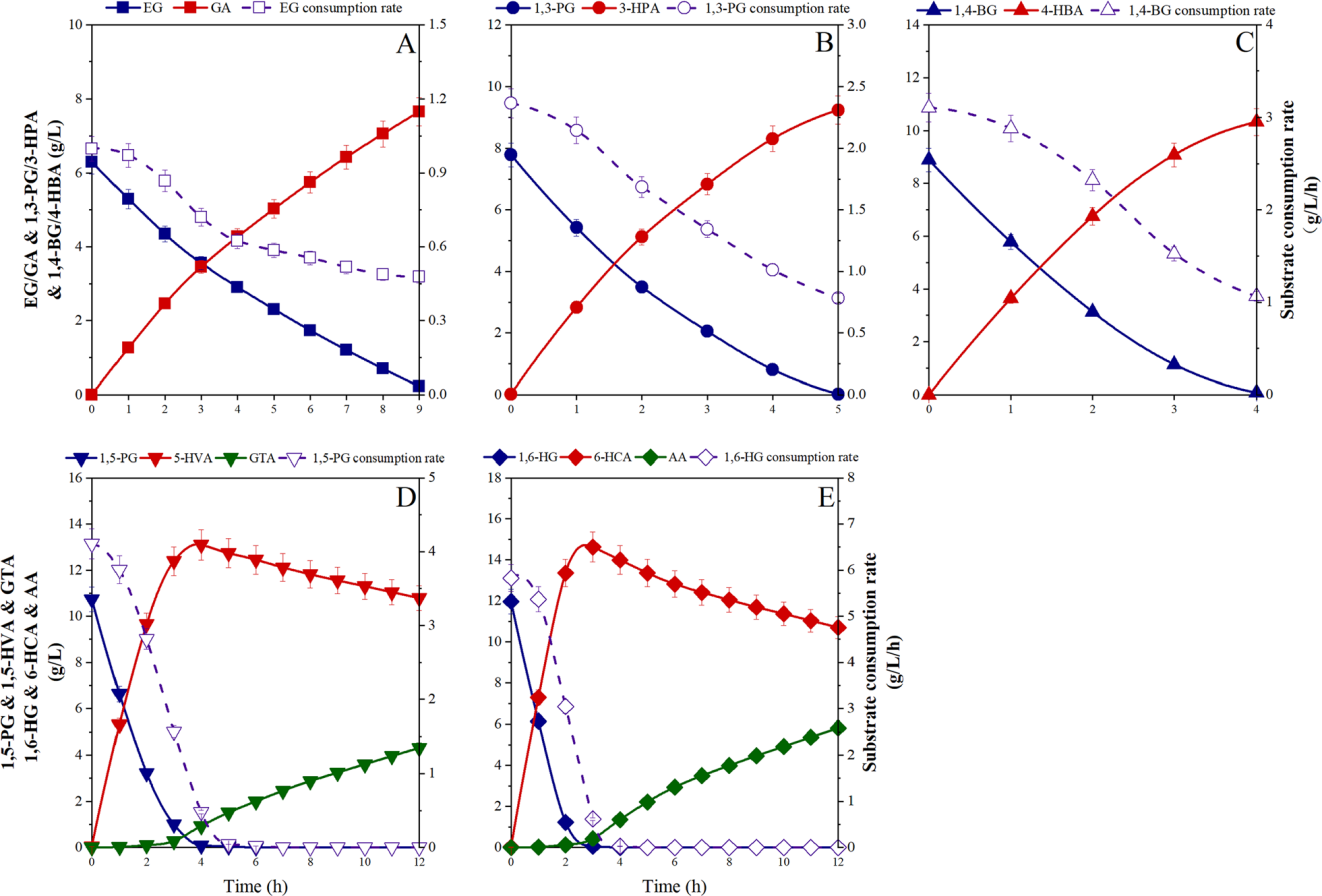
Whole-cell catalysis of diols by *G. oxydans* NL71 with pH control at 5.5. **A**: EG; **B**: 1,3-PG; **C**: 1,4-BG; **D**: 1,5-PG; **E**: 1,6-HG. Line: diol content (blue); accumulated hydroxyl acid content (red); accumulated diacid content (green); diol consumption rate (purple)

**Fig. 3:**
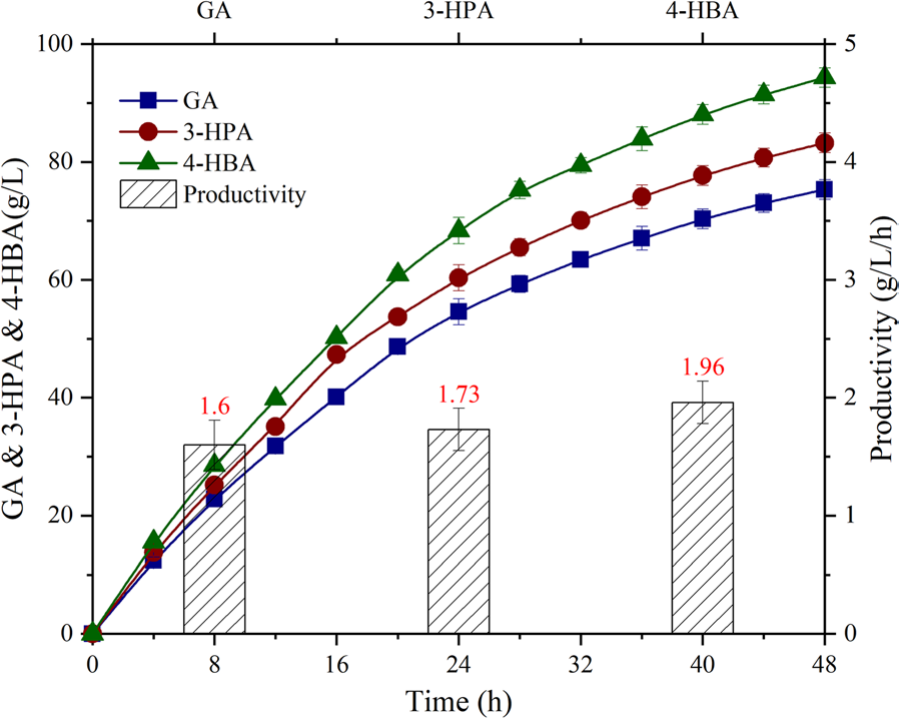
Comparison of the whole-cell catalysis for GA, 3-HPA and 4-HBA production by *G. oxydans* with SOS technology. The symbols indicated accumulated GA content (■), and accumulated 3-HPA content (●) and accumulated 4-HBA content (▲) in the broth. The columns represented the average productivity of the three hydroxyl acids

Dual-functional modules of hydroxyl acids alleviate their application prospects in many high-end fields [44]. However, the traditional hydroxyl acid preparation methods would produce abundant of wastes and by-products, violating the principles of green chemistry and sustainable development. Hence, employing *G. oxydans* as a core catalyst to design a green and environmental-friendly hydroxyl acids synthesis method is an effective approach to solve the current industrial bottlenecks. To explore the reaction mechanism of *G. oxydans* whole-cell catalysis, five linear diols were selected as substrates for kinetic study with OD_600_ = 2. Figure 2 depicts the bioprocesses for the catalysis of EG, 1,3-PG, and 1,4-BG by *G. oxydans*. The final catalytic products were GA, 3-HPA, and 4-HBA, respectively, without the formation of diacids. The average consumption rates of EG, 1,3-PG, and 1,4-BG were 0.68, 1.55, 2.23 g/L/h, respectively. Compared with the chemical method, the purity of products was satisfactory, meeting the core requirements of green chemistry, and the HPLC chromatograms in Additional file 1: Figures S1, S2 and Fig. 3. However, the reaction efficiency still needed to be improved to meet the requirements for industrial production.

Figure 2 shows the biocatalysis of 1,5-PG and 1,6-HG by *G. oxydans,* respectively. Surprisingly, when the carbon chain length was ≥ 5, the end products showed qualitative differences. 1,5-PG and 1,6-HG catalysis followed a step-by-step process, further catalyzing to GTA and AA when primary diols were oxidized to hydroxyl acids. Moreover, the catalytic rates for 1,5-PG and 1,6-HG were very similar. The substrate consumption time was less than 3 h and 4 h, while the average substrate consumption rates reached 3.25 and 5.38 g/L/h, respectively. Although the reaction efficiency of C5/C6 was promising, the product quality was not satisfactory due to the formation of by-products (diacids). At present, hydroxyl acids have attracted much attention due to their dual-functional modules, which exhibit excelling properties. Hence, we need further explored directional regulation for hydroxyl acids production by *G. oxydans* to lay a theoretical foundation for the industrial production.

### The whole-cell catalysis of primary diols (C2–C4) by *G. oxydans* combined with SOS technology

Previous studies indicated that *G. oxydans* could bio-transform EG, 1,3-PG, and 1,4-BG into hydroxyl acids. Although the purity of the products met the green chemistry and industrial production requirements, the catalytic efficiency was low to strengthen the production economy. *G. oxydans* takes oxygen as the final electron receptor, so the whole-cell catalysis requires a lot of oxygen carriers to enhance the catalytic rate [45, 46]. However, the high cost of unrestricted oxygen supply cannot be borne by the industry. Therefore, in this study, SOS technology was employed to maximize oxygen utilization while ensuring high catalytic efficiency. Moreover, SOS technology safely controls the pressure in the bioreactor by adjusting the oxygen inlet speed, eliminating the potential safety hazards in industrial production [43].

As shown in Fig. 3, 48 h whole-cell catalysis of EG, 1,3-PG, and 1,4-BG were carried out in SOS-BR with *G. oxydans* OD_600_ = 2. Results indicated that GA, in the 48 h bioprocess, 3-HPA and 4-HBA accumulated 75.3 g/L, 83.2 g/L, and 94.3 g/L respectively. In terms of GA production, the highest quantity of GA obtained was 74.5 g/L with a productivity of 1.49 g/L/h by Wei et al. [47], which was lower than 1.6 g/L/h obtained in this study. Sun et al. reported a recombinant *Escherichia coli* that produce 38.7 g 3-HPA with an average yield of 35% in 72 h fermentation [48], the productivity was lower than SOS technology and numerous by-products were found. For 4-HBA, Sang et al. [49] employed recombinant *Escherichia coli* to produce 103.4 g/L under the microaerobic conditions with a productivity of 0.844 g/L/h. Although the concentration of 4-HBA was slightly higher than that of whole-cell catalysis, its productivity was only 43% of SOS technology. Furthermore, a lot of inhibitors such as acetic acid were generated in the fermentation broth of recombinant *Escherichia coli*, which seriously affected the product quality. Apparently, the whole-cell catalysis with *G. oxydans,* combined with SOS technology has realized the continuous and efficient preparation of high-purity hydroxyl acids (C2–C4), which provided solid technical support for their industrial production.

### Bioprocess for 5-HVA preparation with pH regulation and cell-recycling technology in SOS-BR

**Fig. 4:**
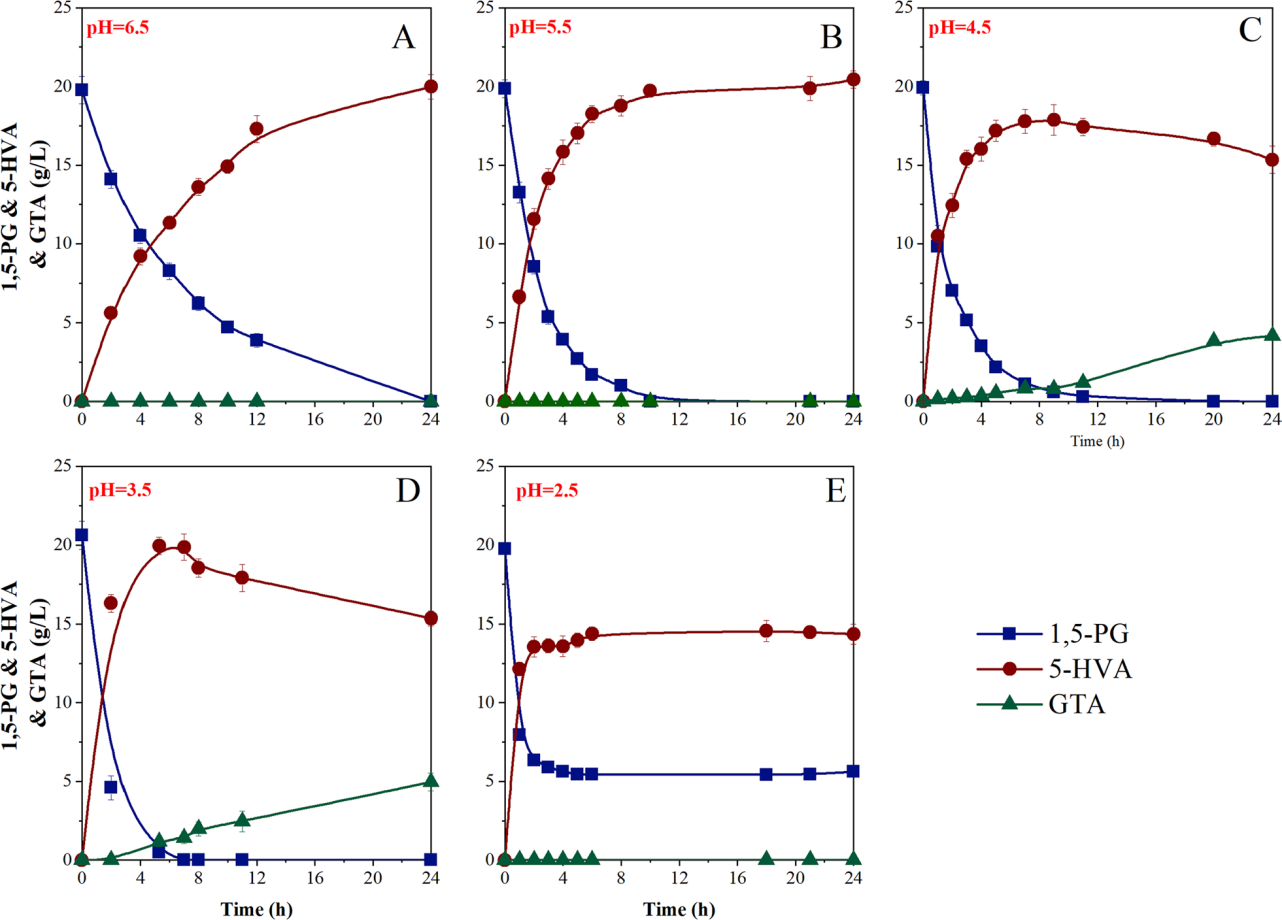
Comparison of the whole-cell catalysis of 1,5-PG by *G. oxydans* with different pH regulation. **A**: pH = 2.5; **B**: pH = 3.5; **C**: pH = 4.5; **D**: pH = 5.5; E: pH = 6.5. The symbols indicated the concentration of 1,5-PG (■), and accumulated 5-HVA content (●) and accumulated GTA content (▲) in the reaction

A previous study showed that 5-HVA could be produced by *G. oxydans*, its production level was rather unsatisfactory, and GTA was accumulated in the system as a by-product. Moreover, the traditional chemical methods cannot prepare high-quality 5-HVA due to high cost, which limits their application in advanced fields such as medicine and material science. Therefore, targeted regulation for selective catalysis of 1,5-PG is a promising approach for the industrial production. As shown in Fig. 4, the whole-cell catalysis of 1,5-PG was performed under different pH gradients, including pH = 2.5, 3.5, 4.5, 5.5 and 6,5. When pH ≥ 5.5, GTA was not produced even if the substrate 1,5-PG was completely bio-oxidized to 5-HVA. However, when pH value was less than 5.5, the whole-cell catalysis showed two-stage reactions; 1,5-PG generated 5-HVA in the first step, and then 5-HVA was catalyzed to GTA in the second step. It is noteworthy that the results at pH = 2.5 were contrary to the law, because normal physiological activity could not be maintained under extremely acidic conditions, and *G. oxydans* lost catalytic activity after 2 h. Therefore, the proposed scheme of pH-regulated whole-cell catalysis provided a green and high-quality approach for the industrial production of 5-HVA without any by-products. To directionally obtain an ultra-high titer of 5-HVA, we selected pH = 5.5 for the bioreactor experiment.

**Fig. 5:**
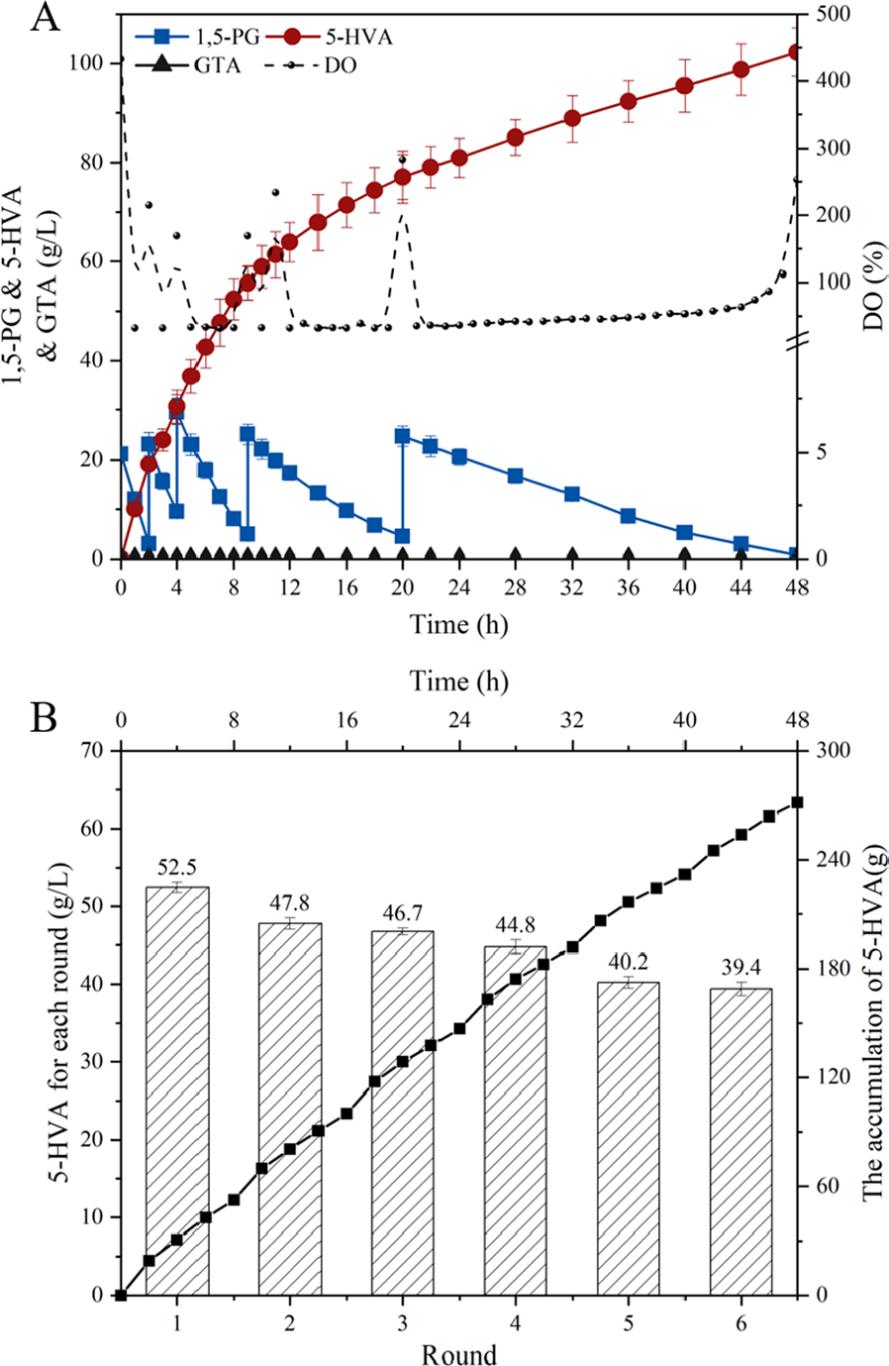
Whole-cell catalysis of 1,5-PG by *G. oxydans* with pH control at 5.5. **A**: SOS technology, the symbols indicated the concentration of 1,5-PG (■), accumulated 5-HVA content (●), accumulated GTA content (▲) and the dotted line represented the DO level. **B**: cell recycling technology, the symbols indicated the accumulation of 5-HVA (■) and the columns represented the 5-HVA production achieved in each round

In 2019, Keiichi et al. employed over-supported platinum catalysts to produce 5-HVA, δ-valerolactone, and methyl 5-hydroxyvalerate with the yield of 62% [17]. The results revealed that the yield was low and there were abundant of derivatives, which seriously affected the purity of products. Moreover, in 2021, Hee et al. reported the fermentative production of 5‑HVA by metabolically engineered *Corynebacterium glutamicum*, and 55 g/L 5-HVA and 10 g/L GTA were produced after 28 h fermentation [50]. Apparently, their designed bioprocess was not suitable for industrial-scale production of 5-HVA. Hence, we conducted whole-cell catalysis for 5-HVA bio-production in SOS-BR with pH regulation at 5.5 (Fig. 5A). Results showed that 102.3 g/L of 5-HVA was accumulated without the formation of diacids during 48 h whole-cell catalysis with average productivity of 2.1 g/L/h, and the HPLC chromatograms was shown in Additional file 1: Figure S4. Simultaneously, the production in the first 10 h was 58.9 g/L and the productivity was 5.9 g/L/h, exceeding the highest level of 5-HVA (55 g/L during 28 h). The fermentation broth contained only 0.2 g/L substrates, without any diacid production at 48 h, and 5-HVA yield was as high as 99.4%. In addition, SOS-BR maintained a high dissolved oxygen (DO) level during the whole-cell catalysis process to meet the oxygen demand for *G. oxydans*. At the same time, due to the strict sealing environment of the entire system, the cost of oxygen utilization was greatly saved and the economy of the entire bioprocess was improved. Moreover, we successfully performed cell-recycling technology with 6 rounds in SOS-BR (Fig. 5B). During 48 h whole-cell catalysis, a total of 274.1 g 5-HVA was accumulated, and the production of each round was 52.5 g/L, 47.8 g/L, 46.7 g/L, 44.8 g/L, 40.2 g/L, and 39.4 g/L, respectively. In conclusion, combined with pH control and SOS-BR technology, we successfully synthesized 5-HVA with high-quality and ultra-high titer, which provided a promising avenue for the industrialization of 5-HVA.

### Bioprocess for 6-HCA synthesis with pH regulation and cell-recycling technology in SOS-BR

**Fig. 6:**
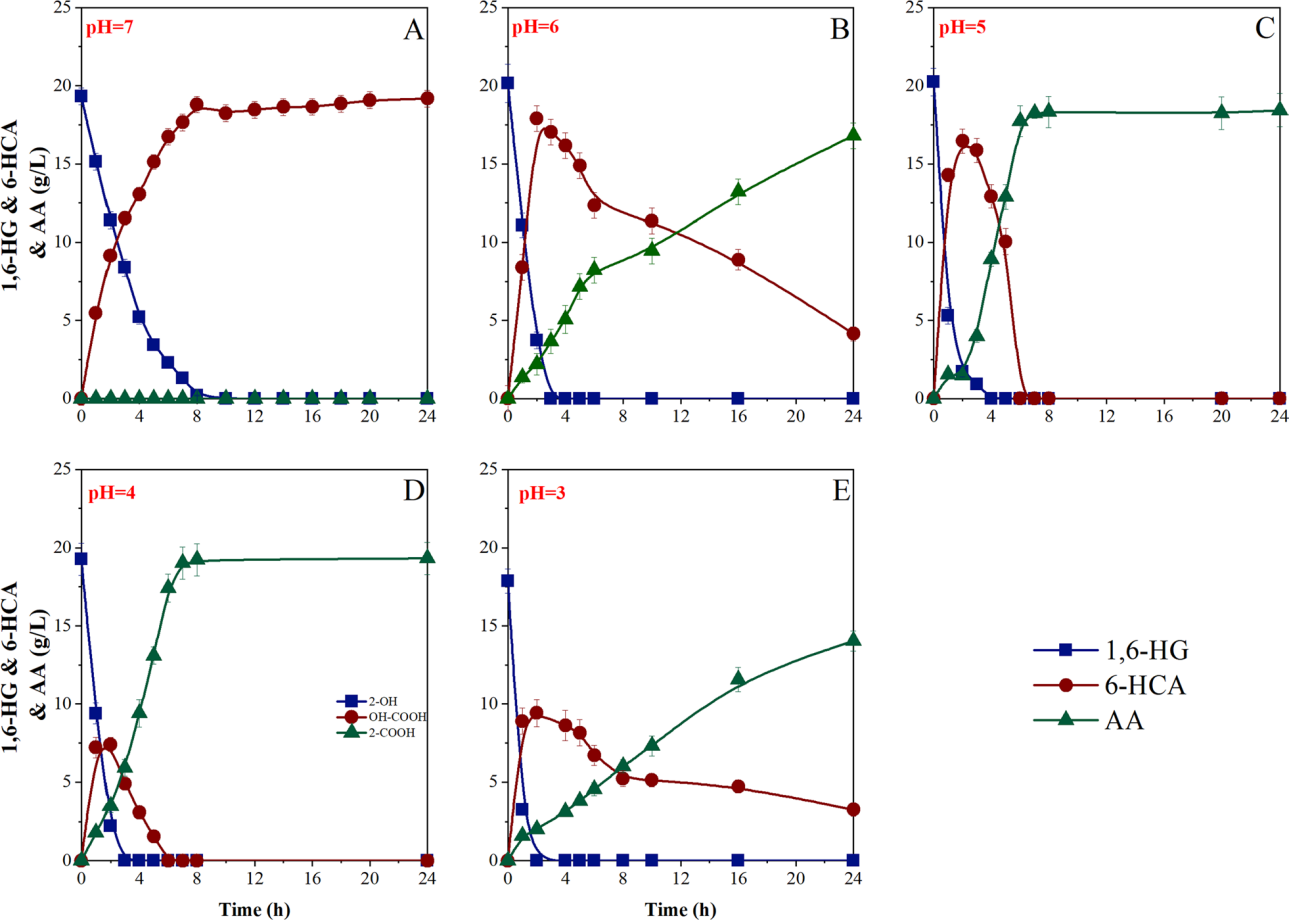
Comparison of the whole-cell catalysis of 1,6-HG by *G. oxydans* with different pH regulation. **A**: pH = 3; **B**: pH = 4; **C**: pH = 5; **D**: pH = 6; **E**: pH = 7. The symbols indicated the concentration of 1,6-HG (■), and accumulated 6-HCA content (●) and accumulated AA content (▲) in the reaction

Compared with other hydroxyl acids, the industrial production technology of 6-HCA is currently unavailable. As 6-HCA is an intermediate, hydroxyl and carboxyl groups often undergo oxidation or reduction during synthesis to generate 1,6-HG, AA, and other by-products. At present, there is a little literature on the preparation of 6-HCA. In 1999, Fischer et al. employed metal catalysts to prepare 6-HCA at high temperature (100 ~ 300℃) and high pressure (10 ~ 300 bar), but the products were accompanied by abundant 1,6-HG and esters [51]. Therefore, it was significant to develop a green and efficient synthesis of 6-HCA by *G. oxydans*. Results revealed that the pH value of fermentation broth had an obvious regulatory effect on the whole-cell catalytic process (Fig. 6). At pH ≤ 6, the whole-cell catalysis of 1,6-HG was divided into two stages. The first step was to oxidize 1,6-HG to an intermediate product 6-HCA, and then convert it to AA. Surprisingly, when the pH of broth was adjusted to 7, the conventional two-stage catalysis was regulated at the first stage. The results suggested that when 1,6-HG was oxidized to 6-HCA, it would not further react to form AA, improving the product quality of 6-HCA and eliminating the formation of by-products. According to previous research results, the weak acidic environment was more suitable for the physiological and biochemical capacity of *G. oxydans*, hence, pH = 7 was selected for 6-HCA biopreparation.

**Fig. 7:**
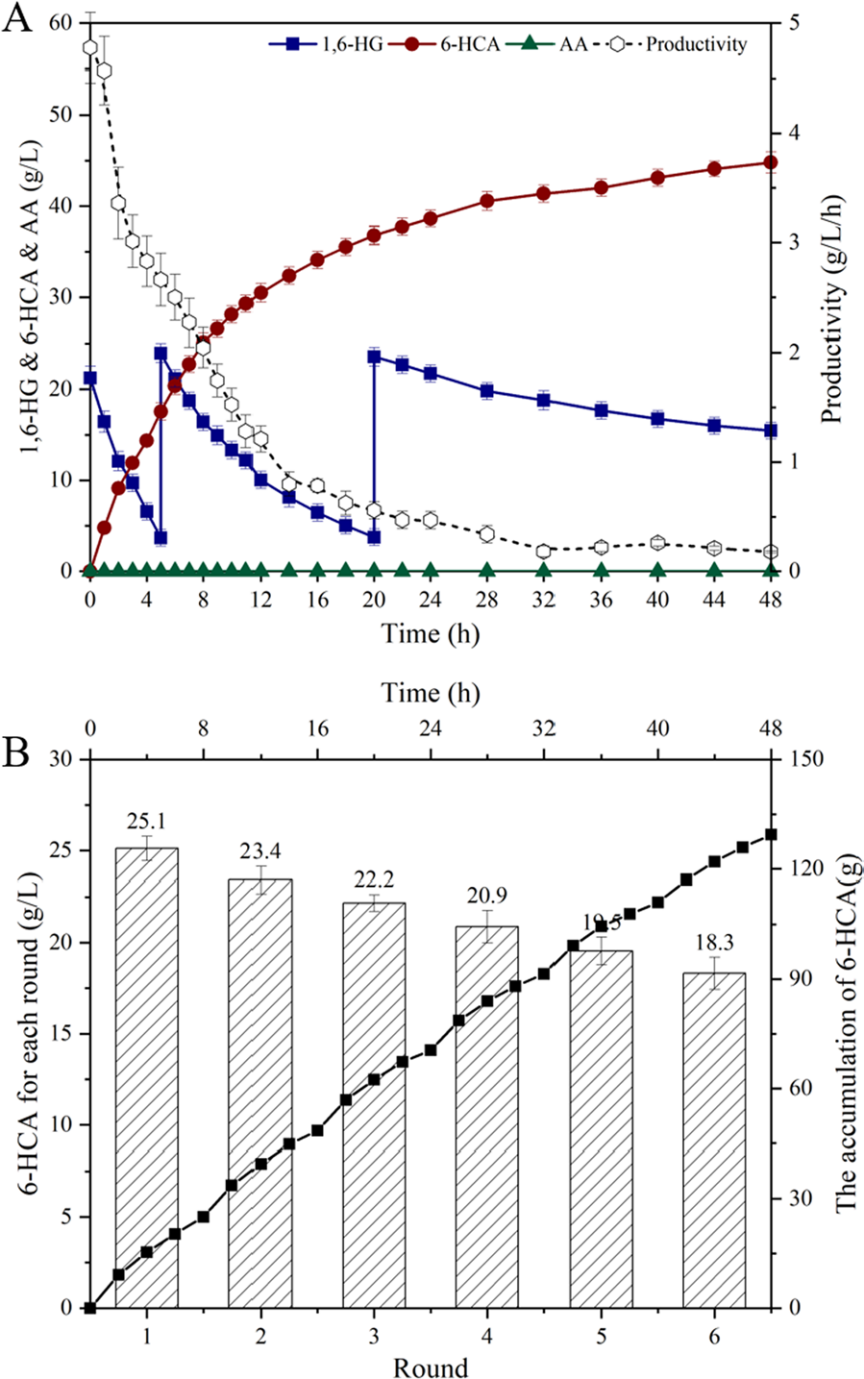
Whole-cell catalysis of 1,6-HG by *G. oxydans* with pH control at 7. **A**: SOS technology, the symbols indicated the concentration of 1,6-HG (■), accumulated 6-HCA content (●), accumulated AA content (▲) and the dotted line represented the DO level. **B**: cell recycling technology, the symbols indicated the accumulation of 6-HCA (■) and the columns represented the AA production achieved in each round

According to the results of pH regulation experiment, the whole-cell catalysis for the preparation of 6-HCA was performed at pH = 7 (Fig. 7A). In fed-batch mode, 48.8 g/L of 6-HCA with the yield of 68.7% was accumulated within 48 h, and the productivity was 1.01 g/L/h, which was the highest reported level for 6-HCA biopreparation. Moreover, the HPLC chromatogram was shown in Additional file 1: Figure S5. Compared with Sang-Hyun et al., the productivity increased 188.6% from 0.35 g/L/h to 1.01 g/L/h [41]. The kinetic curve showed that the inhibition effect was evident after the formation of hydroxyl acids and the productivity decreased from 4.5 g/L/h to 0.3 g/L/h. When the catalysis was performed for 8 h, the productivity decreased to 2 g/L/h, 50% lower than that of the initial level. Therefore, we performed 6 rounds of cell-recycling experiment for single batch catalysis, and the results are shown in Fig. 7B. During 48 h of whole-cell catalysis, the cell-recycling experiment was successfully implemented for 6 rounds, and the last round still maintained 72.9% capacity. Finally, 129.3 g of 6-HCA was accumulated, with average productivity of 2.7 g/h, which was 1.6 times that of one batch for 48 h continuous catalysis. The importance of 6-HCA underscores its social demands, but the existing technologies cannot support the market demands. Therefore, the preparation method of 6-HCA proposed in the study, overcomes the disadvantages of traditional methods, demonstrating promise for the industrial production of 6-HCA in the future.

## Conclusions

In this study, a microbiological regulation process for high value-added hydroxyl acids synthesis from primary diols by *G. oxydans* was successfully established. Combined with SOS technology, the whole-cell catalytic synthesis of high-titer GA, 3-HPA, and 4-HBA was realized. Further enhancement in 5-HPA and 6-HCA production was successfully realized by pH regulation without any diacids generated. In conclusion, the whole-cell catalysis of diols by *G. oxydans* for industrial-scale preparation of hydroxyl acids was established. Moreover, the mild condition of whole-cell catalysis agrees well with the principles of green synthesis for the environment-friendly production of hydroxyl acids.

## Supplementary Information

**Additional file 1: Figure S1.** HPLC chromatograms of standard sample (4 g/L) and whole-cell catalysis for GA production. **Figure S2.** HPLC chromatograms of standard sample (4 g/L) and whole-cell catalysis for 3-HPA production. **Figure S3**. HPLC chromatograms of standard sample (4 g/L) and whole-cell catalysis for 4-HBA production. **Figure S4.** HPLC chromatograms of standard sample (4 g/L) and whole-cell catalysis for 5-HPA production with pH regulation. **Figure S5**. HPLC chromatograms of standard sample (4 g/L) and whole-cell catalysis for 6-HCA production with pH regulation.

## Data Availability

All data generated and analyzed in this study are included in this published article.
