# Supplementary material for: pH regulatory divergent point for the selective bio-oxidation of primary diols during resting cell catalysis
Source: Biotechnol Biofuels Bioprod. 2022 Jun 30;15:73. doi: 10.1186/s13068-022-02171-5 (PMC9248139; doi:10.1186/s13068-022-02171-5)
Supplement: Supplementary file 1 — Additional file 1: Figure S1. HPLC chromatograms of standard sample (4 g/L) and whole-cell catalysis for GA production. Figure S2. HPLC chromatograms of standard sample (4 g/L) and whole-cell catalysis for 3-HPA production. Figure S3. HPLC chromatograms of standard sample (4 g/L) and whole-cell catalysis for 4-HBA production. Figure S4. HPLC chromatograms of standard sample (4 g/L) and whole-cell catalysis for 5-HPA production with pH regulation. Figure S5. HPLC chromatograms of standard sample (4 g/L) and whole-cell catalysis for 6-HCA production with pH regulation. [file 13068_2022_2171_MOESM1_ESM.docx]

**Figure caption**

**Figure S1.** HPLC chromatograms of standard sample (4 g/L) and whole-cell catalysis for GA production.

**Figure S2.** HPLC chromatograms of standard sample (4 g/L) and whole-cell catalysis for 3-HPA production.

**Figure S3**. HPLC chromatograms of standard sample (4 g/L) and whole-cell catalysis for 4-HBA production.

**Figure S4.** HPLC chromatograms of standard sample (4 g/L) and whole-cell catalysis for 5-HPA production with pH regulation.

**Figure S5**. HPLC chromatograms of standard sample (4 g/L) and whole-cell catalysis for 6-HCA production with pH regulation.

**Figure S1.**





**Figure S2.**





**Figure S3.**





**Figure S4.**





**Figure S5.**
